# Socio-demographic trends in malaria knowledge and implications for behaviour change interventions in Zanzibar

**DOI:** 10.1186/s12936-023-04472-y

**Published:** 2023-02-02

**Authors:** Faiza Abbas, Emmanuel Kigadye, Fauzia Mohamed, Mwinyi Khamis, John Mbaraka, Naomi Serbantez, Abdul-Wahid Al-Mafazy, April Monroe, Samson Kiware

**Affiliations:** 1 Zanzibar Malaria Elimination Programme (ZAMEP), Ministry of Health, Zanzibar, United Republic of Tanzania; 2 The Open University of Tanzania, Dar es Salaam, United Republic of Tanzania; 3 Ifakara Health Institute, Dar es Salaam, United Republic of Tanzania; 4 US President’s Malaria Initiative, U.S. Agency for International Development, Dar es Salaam, United Republic of Tanzania; 5 RTI International, Dar es Salaam, United Republic of Tanzania; 6 Johns Hopkins Center for Communication Programs, Baltimore, MD USA; 7 Pan African Mosquito Control Association (PAMCA), Nairobi, Kenya

**Keywords:** Knowledge on malaria, Local malaria transmission, Incidence, Zanzibar, Tailored SBC, Intervention

## Abstract

**Background:**

Zanzibar is among the few places within East Africa that have documented a significant reduction of malaria morbidity and mortality. Despite tremendous gains over the past decade, malaria transmission still persists in Zanzibar. This study aimed at understanding levels of malaria knowledge to provide recommendations that can be used to reinforce and scale up targeted malaria social and behaviour change interventions.

**Methods:**

A descriptive cross-sectional survey was conducted through an administered questionnaire to 431 households selected randomly. The interviewees were the heads of household or representative adults above 18 years. This study investigated the levels of knowledge about the causes, symptoms, and prevention of malaria in areas with high (> 1.9 per 1000) and low (< 1 per 1000) incidence of local malaria cases. The Principal Component Analysis (PCA) was used to compute the composite variable of each category. Descriptive statistics were calculated to understand variables of interest between low and high transmission areas. Multinomial logistic regression model was used to compare knowledge on malaria based on key variables.

**Results:**

A total of 431 heads of households were interviewed. Respondent age, education level, and wealth status were significantly associated with variations in level of malaria knowledge. Old age was found to be significantly associated with low knowledge of malaria (P < 0.001). The majority of study participants who had secondary and higher education levels had good knowledge of malaria (P < 0.006). Participants characterized as middle-income had good knowledge compared to those characterized as low-income (P < 0.001).

**Conclusion:**

The study identified existing gaps in malaria knowledge in low and high transmission areas. Low levels of malaria knowledge were documented among elderly and populations with lower education and income levels. There is a need to extend mobilization, advocacy, and expand channels of communication to reach all community members. The reported gaps in knowledge are important to consider when designing strategies to engage communities in malaria elimination in Zanzibar. Tailored social and behavioural change interventions aiming to increase malaria knowledge could enhance the uptake of malaria prevention services in the community.

## Background

The World Health Organization (WHO) World Malaria Report 2021 indicates that about 96% of the estimated 241 million malaria cases and 627,000 deaths attributed to malaria globally are reported from countries in Africa [1]. A WHO Global technical strategy for malaria 2016–2030 has been developed to provide technical guidance to countries in scaling up malaria responses and moving towards elimination. The strategy has set ambitious targets of attaining malaria elimination to 10 countries in 2020, 20 countries in 2025, and 35 countries in 2030 [2, 3]. However, globally, malaria targets are off track by 37% at the current trajectory [4]. In addition, Zanzibar has been identified by the WHO and the Southern African Development Community (SADC) as being among those countries eligible for malaria pre-elimination, Zanzibar is expected to attain malaria elimination by 2023, according to the National Malaria Strategic Plan [5].

In Zanzibar, the prevalence of asymptomatic infection in the general population has declined from above 25% in 2005 to less than 1% where it has been maintained since 2008 [6, 7]. Data from health facilities across the 11 districts indicate that the number of confirmed malaria cases declined from 12,000 in 2005 to 3,500 in 2015 [6]. In 2016, the malaria programme strengthened the surveillance activities to classify all confirmed cases where local (indigenous) malaria transmission is defined as any case contracted locally with no strong evidence of a direct link with imported cases [8].

All passively detected malaria confirmed cases are reported from 305 public and private health facilities. District Malaria Surveillance Officers conduct investigations at index cases households to collect information by standardized questionnaire that allows classification of each confirmed malaria case by the origin of infection, i.e., imported, introduced, indigenous, or induced [8].

Despite the tremendous achievements in maintaining low disease prevalence, progress has begun to stall, in recent years the number of reported malaria cases increased from 4,106 in 2018 to 9290 in 2021 [9, 10]. The annual parasite incidence has increased from 2.7 per 1000 population in the strategic plan baseline (2017) to 4.06 in 2019 [11]. Malaria transmission in Zanzibar is unevenly distributed from zone to district level. In 2021, Unguja island contributed to 86% (8033) compared to Pemba that reported only 14% (1300) of the total 9290 reported malaria cases. West B and Urban were the leading districts contributing to 22% and 20% of all reported cases.

The coverage of malaria interventions across the island is high. According to the ZAMEP annual report of 2021 [10], 100% of suspected malaria cases received parasitological test and 100% of all confirmed malaria cases in public health facilities were provided with appropriate treatment. The coverage of targeted indoor residual spraying (IRS) was 94% (n = 46,316), protecting a population of 230,708 (94.5%) and mass long-lasting insecticidal bed nets (LLIN) coverage of 96% [10]. Several factors may have contributed to the malaria increase. It is, therefore, important to understand if lack of knowledge on malaria leading to less individual level participation was among the contributing factors.

Current interventions, including LLINs, IRS, and community case management are effective only if they are accessible, acceptable, and properly used within communities [12]. The WHO reported that having good knowledge of malaria causes, signs and symptoms, mode of transmission, and prevention measures has led to the use of malaria prevention strategies and improved health-seeking behaviour [13].

Human behaviour can also play a vital role in reducing malaria transmission and infection [14]. Elimination requires focus on reducing and eliminating malaria transmission foci at a local level and community awareness can improve participation in malaria elimination efforts. These efforts could greatly accelerate the realization and sustainability of the malaria elimination strategy [15]. Knowledge of one’s own disease has often been emphasized as an important cognitive factor that can have a considerable impact on the patient’s adaptation to the disease and on the courses and its treatment [16]. Many factors have been contributing to low coverage and utilization/acceptance of malaria related interventions by the community [17–19]. Several studies have been conducted previously to identify the level of knowledge among different population groups and the reports regarding the levels of malaria knowledge and associated factors differs among various studies. However, the majority of literature concluded that having a good knowledge regarding malaria cause, mode of transmission, sign and symptom, and prevention of malaria can increase the use of malaria prevention interventions and health-seeking behaviour [19, 20].

In Zanzibar, the first Malaria Knowledge, Attitude, Practice and Behaviour (KAPB) survey was conducted in 2014 followed by the second in 2017. The Malaria KAPB 2017 study sought to determine levels of knowledge, attitudes and practices about malaria in both rural and urban residents and contribute to development of the knowledge framework for developing new intervention strategies for malaria elimination in Zanzibar [21]. Despite the useful information revealed from the previous survey, there are a number of subjects that need recent information. It is important to understand the current situation regarding the overall malaria knowledge especially in areas with low and high malaria transmission. The objective of this study was to determine the level of malaria knowledge on causes, symptoms and prevention among *shehia* (lowest administrative structure) (the term shehia can be used in the plural, i.e. shehias) with a high and low incidence of local malaria. This could guide the malaria programme with the development of targeted social behavioural change (SBC) interventions based on current existing gaps in malaria knowledge in low and high transmission shehias. This study only focused on knowledge rather than considering all possible psychosocial factors that can influence behaviour [22] many previous studies conducted elsewhere indicate that when knowledge on disease is high—community members' acceptance and utilization of health services increase as well [19, 20].

## Methods

### Study design and the sample size

Zanzibar is a semi-autonomous part of the United Republic of Tanzania, consisting of numerous small islands and two larger populated islands (Unguja and Pemba) in the Indian Ocean. Zanzibar is organized by 5 regions, 11 districts, and 387 shehias.

**Fig. 1:**
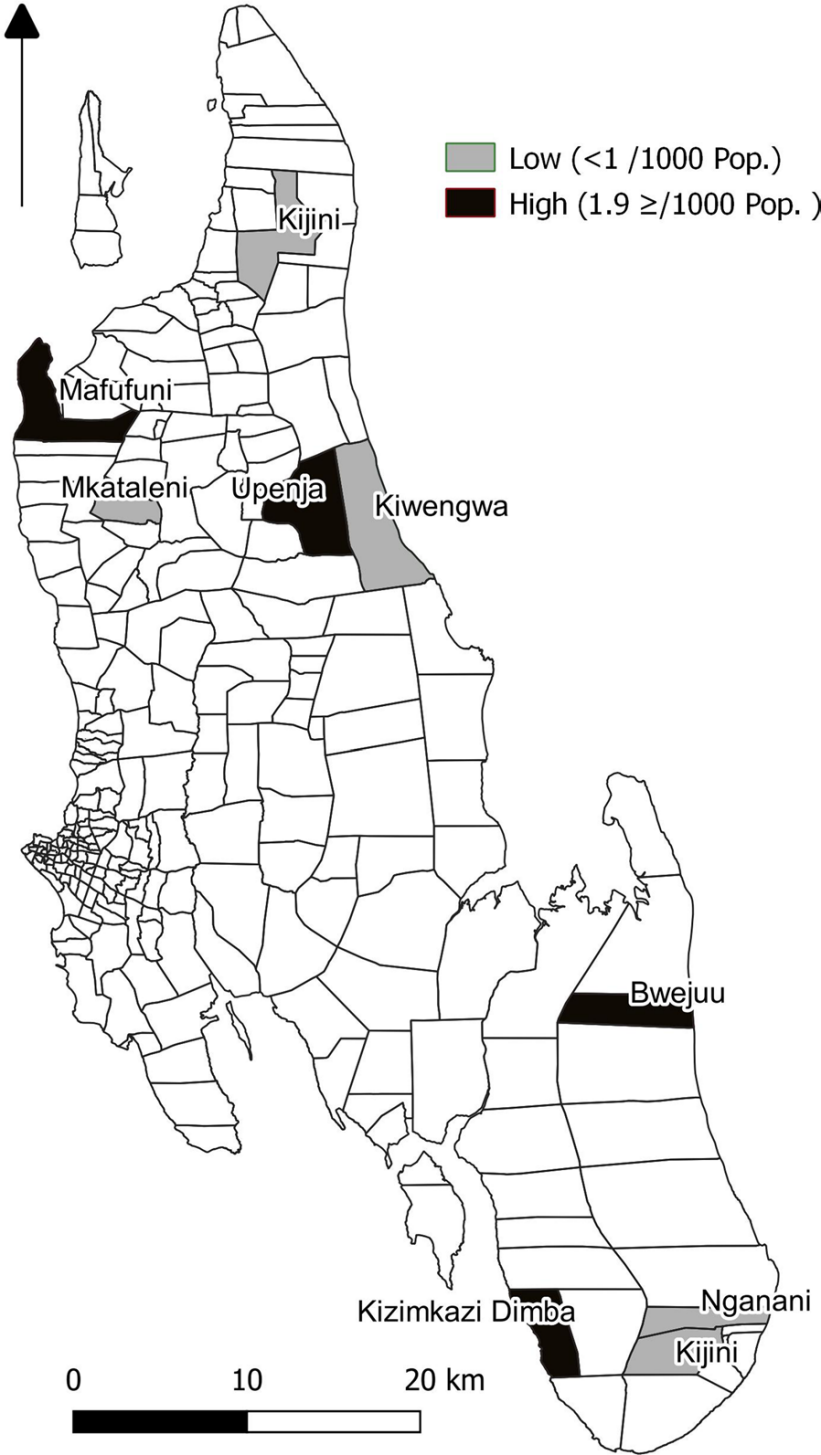
Sampled shehias with high and low incidence of locally acquired, confirmed malaria cases in Unguja, 2019

This study was a descriptive cross-sectional survey conducted in two districts, Kusini and Kaskazini B, in Unguja, forming a total of 52 shehias. Through multistage sampling, eight shehias were selected and organized by the incidence of locally acquired, confirmed malaria: four shehias were grouped with an incidence ≥ 1.9 per 1000 population and four shehias were grouped with an incidence < 1 per 1000 population (Fig. 1) indicating high and low transmission shehias, respectively.

A multistage sampling technique was used to select study participants. In the first stage, a random sampling method was used to first select regions and then districts. In the second stage, purposive sampling was used to select enumeration areas (EAs) whereby all shehias were listed, four shehias with the lowest incidence and four shehias with the highest incidence of locally acquired, confirmed malaria cases within the selected districts were identified. During this stage, four shehias with the lowest incidence of local transmission had an incidence of < 1 per 1000 population and four shehias with the highest incidence of local transmission had an incidence of ≥ 1.9/1,000. In the third stage, random sampling was used to select the number of households in each EA (shehia level). The sample size calculation based on 95% confidence interval, 5% absolute precision, and accounting for 10% non-response rate resulted in a total of 422 households. However, the random selection process resulted in 431 households that were then included in this study. So, a total of 431 participants from 8 shehias aged 18–60 + years were interviewed in this household survey, with 213 households in low incidence shehias and 218 households in high incidence shehias.

### Data collection

Structured questionnaires were designed to seek information on participant demographic information, education level, wealth determinants, and knowledge about the causes, symptoms, and prevention of malaria. All heads of the household or his/her representative ages > 18 years were interviewed. Only one person per household was interviewed. The questionnaire was adapted from the previous knowledge, attitudes, and practices (KAP) studies [12, 13, 20]. Participants responded freely to all the questions and they were able to indicate more than one response with an option to specify other response(s) or to respond “I do not know”. The questions and possible structured responses for each variable were as follows:

#### • Knowledge about causes of malaria

The question was: what do you think is the cause of malaria? The following were possible answers: (i) Mosquito bites; (ii) Eating dirty food (iii) Drinking dirty water (iv) Getting soaked with rain (v) Cold or changing weather (vi) Witchcraft/devil spirit (vii) other, specify; and (viii) I don’t know.

#### • Knowledge about common symptoms of malaria

The question was: What are some of the symptoms of malaria? The following were possible answers: (i) fever; (ii) chills; (iii) headache; (iv) joint pain; (v) loss of appetite; (6) Body pain; (vii) Seizure/convulsions; (viii) Not able to eat; and (ix) I don’t know.

#### • Knowledge about malaria prevention

The question was: How can one prevent himself from getting malaria? The following were possible answers: (i) sleeping under a mosquito net; (ii) sleeping under an insecticide-treated bed net; (ii) using mosquito repellent; (iv) avoiding mosquito bites; (v) taking prevention medication; (vi) spraying the house with insecticide; (vii) cutting the grass around the house; (viii) filling in puddles/stagnant water; and (ix) others, specify.

### Data analysis

Completeness of data was ensured by a field supervisor who certified that all questions were administered and all responses were recorded. Completed questionnaires were coded and entered into Statistical Package for the Social Sciences (SPSS)^®^ version 20 to prepare data for analysis. The statistical analysis was conducted using R statistical package version 4.1.3 [23].

### Overall score for malaria knowledge

The questions from three domains had possible multiple responses which are causes of malaria (8 responses), symptoms of malaria (9 responses), and prevention of malaria (9 responses). The wrong answers for each domain were scored 0 and 1 for the correct answers. Total score was calculated for each domain, the cut-off point to understand whether the individual knew a particular domain of malaria was computed using principal component analysis [24] which indicated 0 (poor knowledge) and 1 (good knowledge). The overall scores for malaria knowledge were assessed by combining the three main categories of knowledge related variables, (i) causes; (ii) symptoms; and (iii) prevention measures. An overall knowledge score was calculated by the sum of all the knowledge category scores. Participants who scored 3 were categorized as having high knowledge; those who scored 2 were classified as having medium knowledge, and participants who scored 1 or zero were classified as having low knowledge.

A chi-square test was used to determine the association between sociodemographic characteristics and knowledge score with statistical significance declared at P-value < 0.05. A multinomial logistic regression model was used to determine the three-level outcome variable, low, medium, and high levels of knowledge. Wealth index was calculated based on participants household’s assets—where middle-income households were categorized based on the presence of corrugate iron sheets (roofing), house with windows structure where all windows close, own mobile phone, use cement floor material or tiles, own television, own radio, house electricity, use tap water and use of flash system in toilets. Participants using less of these assets were categorized as low-income.

## Results

### Sociodemographic characteristics of study participants by malaria transmission

Table 1 shows the sociodemographic characteristics of the study participants by malaria transmission level. As indicated in Table 1, the study participants consisted of 107 (25%) males and 324 (75%) females. The majority of participants living in high malaria transmission shehias were females (55.9%) compared to males (P < 0.001). The majority of participants who had been living in their household for five or more years were living in low transmission shehias compared to those living in their household fewer than five years (P = 0.029) (Table 1).

**Table 1:** Sociodemographic characteristics of participants by malaria transmission

Variable	Categories	Malaria incidence (per 1000 population)		Total (N = 431)	P-value
		High (≥ 1.9/1000) (n = 218)	Low (< 1/1000) (n = 213)		
Gender	Female	181(55.9)	143(44.1)	324	< 0.001
	Male	37(34.6)	70(65.4)	107	
Age group	< 30	39(62.9)	23(37.1)	62	0.062
	30–49	89(45.9)	105(54.1)	194	
	≥ 50	90(51.4)	85(48.6)	175	
Education level	No formal	57(48.3)	61(51.7)	118	0.843
	Primary	101(51.3)	96(48.7)	197	
	Secondary and higher	60(51.7)	56(48.3)	116	
Wealth index^b^	Middle-income	101(49.5)	103(50.5)	204	0.745
	Low-income	117(51.5)	110(48.5)	227	
Occupation	Entrepreneur	36(51.4)	34(48.6)	70	0.817
	Formal employment^a^	8(44.4)	10(55.6)	18	
	Farming	120(50.4)	118(49.6)	238	
	Fishing	27(46.6)	31(53.4)	58	
	Other	27(57.4)	20(42.6)	47	
Years living in current shehia	≤ 1	15(68.2)	7(31.8)	22	0.029
	2–5	15(71.4)	6(28.6)	21	
	≥ 6	188(48.5)	200(51.5)	388	

^a^Formal employment refers to an individual hired as an employee under an established contract agreement with a monthly salary including civil servants and those working in the private sector

^b^Wealth index was computed based on ownership of households’ assets listed in our questionnaires with low and medium income categorized as described above

### Characteristics of the study participants by level of malaria knowledge

**Fig. 2:**
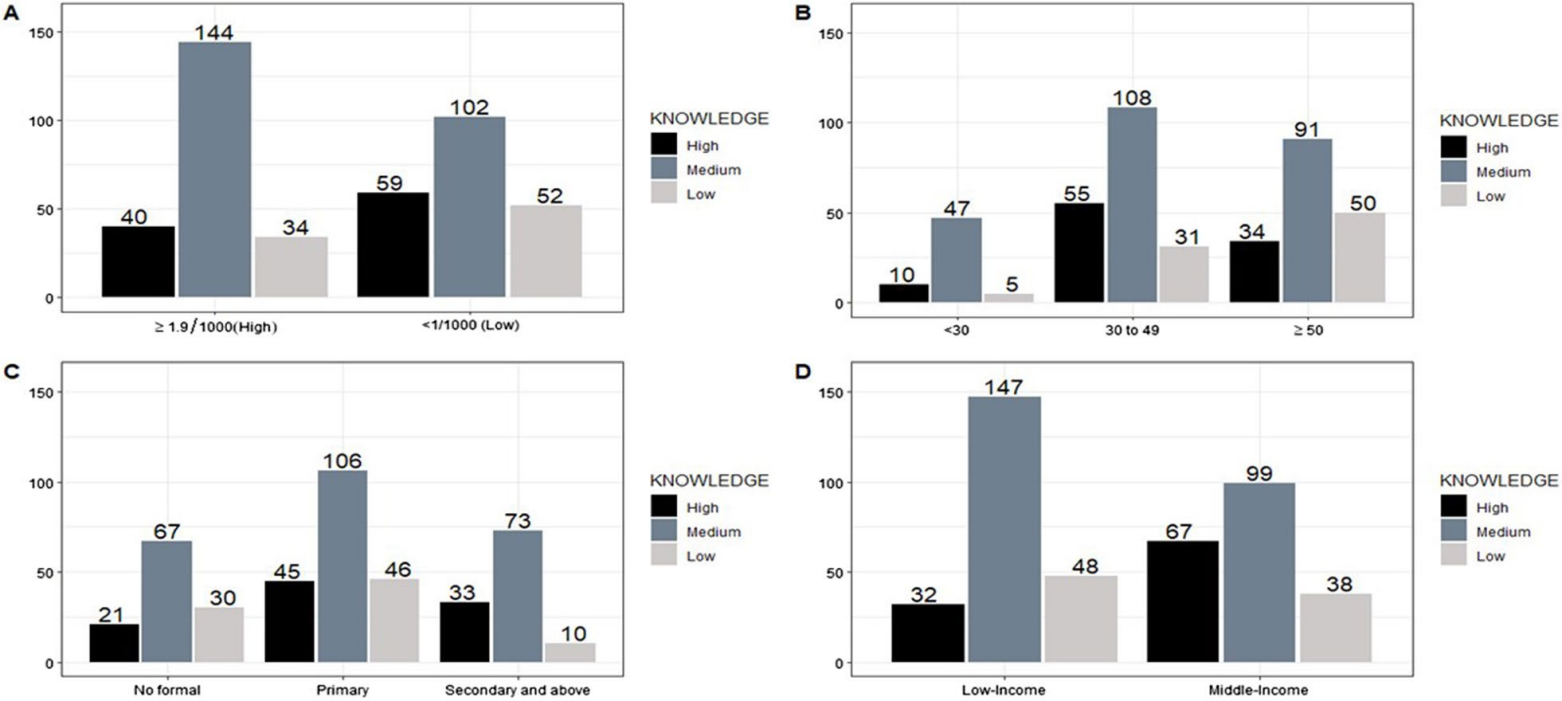
The association between sociodemographic characteristics and malaria knowledge; Frequency of malaria knowledge level by transmission levels (**A**), age groups (**B**), education levels (**C**), and wealth index (**D**)

The majority of participants from low transmission shehias had high knowledge of malaria 59 (27.7%) compared with those living in high malaria transmission 40 (18.4%). The majority of participants with medium knowledge were living in high transmission shehias 144 (66.1%) compared with those living in low malaria transmission 102 (47.9%) (Fig. 2A). Participants in age group 30–49 years had greater high and medium knowledge compared to other age groups (Fig. 2B). Participants with secondary and above education level had high knowledge on malaria 33 (28%) compared to those with primary 45 (23%), and participants with no formal education 21(17%), the majority of them were also having medium knowledge compared to those with primary and no formal education (Fig. 2C). Participants with middle income 67 (32.8%) had a greater high level of malaria knowledge compared to those with low income 32 (14.1%) (Fig. 2D).

Table 2 shows the association between sociodemographic characteristics and malaria knowledge. Participants with low malaria knowledge were more likely to be aged fifty years and older (P < 0.001). Participants who had secondary and above levels of education had higher knowledge of malaria compared with those who had no formal education (P < 0.006). Furthermore, the participants with middle-income wealth had higher knowledge compared to those in the lower-class of wealth (P < 0.001). Participants who were formal employees had higher knowledge compared with farmers and fishermen but not statistically significance (P = 0.051). The majority of participants from high transmission shehias had medium knowledge (66.1%) compared to participants from low transmission shehias (47.9%), participants living in low malaria transmission areas had a greater percentage in the high knowledge category 59 (27.7%) compared with high malaria transmission (18.3%) (P < 0.001).

**Table 2:** Association between sociodemographic characteristics and malaria knowledge

Variable	Categories	Malaria knowledge			Total	P-value
		High (%)	Medium (%)	Low (%)		
Gender	Female	79(24.4)	186(57.4)	59(18.2)	324	0.207
	Male	20(18.7)	60(56.1)	27(25.2)	107	
Age group	< 30	10(16.1)	47(75.8)	5(8.1)	62	< 0.001
	30–49	55(28.4)	108(55.7)	31(16.1)	194	
	≥ 50	34(19.4)	91(52.0)	50(28.6)	175	
Education level	No formal	21(17.8)	67(56.8)	30(25.4)	118	0.006
	Primary	45(22.8)	106(53.8)	46(23.4)	197	
	Secondary and higher	33(28.4)	73(62.9)	10(8.6)	116	
Wealth index	Middle-income	67(32.8)	99(48.5)	38(18.6)	204	< 0.001
	Low-income	32(14.1)	147(64.8)	48(21.1)	227	
Occupation	Entrepreneur	14(20.0)	47(67.1)	9(12.9)	70	0.051
	Formal employment	6(33.3)	10(55.6)	2(11.1)	18	
	Farming	56(23.5)	122(51.3)	60(25.2)	238	
	Fishing	9(15.5)	39(67.2)	10(17.2)	58	
	Other	14(29.8)	28(59.6)	5(10.6)	47	
Years living in current shehia	≤ 1	3(13.6)	15(18.2)	4(18.2)	22	0.144
	2–5	2(9.5)	17(81.0)	2(9.5)	21	
	≥ 6	94(24.2)	214(55.2)	80(20.6)	388	
Malaria incidence (Transmission)	High (≥ 1.9/1000)	40(18.3)	144(66.1)	34(15.6)	218	< 0.001
	Low (< 1/1000)	59(27.7)	102(47.9)	52(24.4)	213	

#### Knowledge on causes, symptoms, and prevention of malaria

The majority of participants had high to medium knowledge on the cause, symptoms, and prevention of malaria. Regardless of malaria transmission shehia, 93% of participants could recognize a mosquito bite as the cause of malaria, 81% associated fever as a symptom of malaria, and 89% identified sleeping under an insecticide-treated bed net as one of the best ways to prevent malaria. However, the majority of the participants who recognized a mosquito bite as the cause of malaria and fever as a common symptom for malaria also incorrectly identified other causes, symptoms and prevention measures.

A narrow majority of participants (51.9%) living in low malaria transmission shehias had significantly higher knowledge on the cause of malaria compared to those living in high malaria transmission shehias (P < 0.001) (Table 3). The majority of participants living in low malaria transmission shehias correctly identified the symptoms associated with malaria and malaria prevention measures compared to those living in high malaria transmission shehias (P < 0.05). The majority of participants living in low malaria transmission shehias were more likely to be aware of the different options to prevent malaria than those living in high transmission shehias (P < 0.05) (Table 3).

**Table 3:** Participant knowledge about causes, symptoms, and prevention of malaria by transmission

Questions/responses	Malaria incidence (per 1000 population)		Total (N = 431)	P-value
	High (%) (≥ 1.9/1000)	Low (%) (< 1/1000)		
*Causes of malaria*				
Mosquito bite	192(48.1)	207(51.9)	399	< 0.001
Eating dirty food	7(29.1)	17(70.8)	24	0.05
Drinking dirty water	2(4.5)	42(95.5)	44	< 0.001
Getting soaked with rain	1(50.0)	1(50.0)	2	1.000
Cold weather	3(33.3)	6(66.7)	9	0.478
Witchcraft/devil sprit	0(0.0)	3(100.0)	3	0.238
Don’t know	19(90.5)	2(9.5)	21	< 0.001
*Malaria symptoms*				
Fever	151(43.5)	196(56.5)	347	< 0.001
Chill	21(18.3)	94(81.7)	115	< 0.001
Headache	105(40.4)	155(59.6)	260	< 0.001
Joint pain	43(37.7)	71(62.3)	114	0.001
Loss of appetite	20(42.6)	27(57.4)	47	0.311
Body pain	49(53.8)	42(46.2)	91	0.559
Goes unconscious	3(42.9)	4(57.1)	7	0.975
Seizure/convolution	9(28.1)	23(71.9)	32	0.014
Not able to eat	42(75.0)	14(25.0)	56	< 0.001
*Prevention measures*				
Sleep under bed net	179(46.9)	203(53.1)	382	0.004
Use of repellant	19(48.7)	20(51.3)	39	0.939
Take prevention medication	35(52.2)	32(47.8)	67	0.854
Use insecticide coil	11(35.5)	20(64.5)	31	0.119
Use door and window netting	27(35.5)	49(64.5)	76	0.005
Bur local plants	2(11.1)	16(88.9)	18	0.001
Wea long sleeves	15(83.3)	3(16.7)	18	0.009

#### Factors associated with malaria knowledge using the multinomial logistic regression model

Table 4 shows the results of regression analysis, which compared sociodemographic characteristics of participants between those with medium to low knowledge, and between those with high to low knowledge.

**Table 4:** The adjusted odds ratio estimates using multinomial logistic regression

Low knowledge (1)	Medium knowledge		High knowledge	
Variable	AOR (95% CI)	P value	AOR (95% CI)	P value
Gender				
Male	1	1	1	1
Female	1.14(0.63–2.04)	0.672	1.81(0.87–3.74)	0.11
Age group				
Less than 30	1	1	1	1
30 to 49	0.51(0.18–0.75)	0.207	0.83(0.24–2.83)	0.798
50 and above	0.26(0.09–0.75)	0.013	0.32(0.09–1.10)	0.07
Education category				
No formal	1	1	1	1
Primary	0.89(0.49–1.62)	0.708	1.10(0.53–2.28)	0.808
Secondary and above	2.93(1.28–6.74)	0.011	3.45(1.34–8.89)	0.01
Wealth index				
Low class	1	1	1	1
Middle class	0.92(0.54–1.57)	0.763	2.70(1.44–5.09)	0.002
Occupation status				
Entrepreneurs	1	1	1	1
Fishing	1.78(0.80–4.00)	0.16	1.14(0.42–3.14)	0.793
Business	1.96(0.86–4.48)	0.111	1.52(0.58–4.01)	0.398
Formal employed	1.73(0.34–8.72)	0.504	2.32(0.42–12.93)	0.338
Other specify	2.10(0.74–5.92)	0.161	2.46(0.79–7.63)	0.121
Living years				
Less than 2	1	1	1	1
2 to 5	1.91(0.27–13.38)	0.517	1.03(0.08–14.01)	0.963
6 and above	1.06(0.30–3.71)	0.927	1.70(0.32–8.99)	0.534
Transmission status				
Low	1	1	1	1
High	2.21(1.29–3.79)	0.004	1.03(0.54–1.94)	0.936

*AOR* Adjusted odds ratio

#### Medium compared to low knowledge

Participants aged ≥ 50 years were more likely to have low malaria knowledge than younger age groups (adjusted OR = 0.26; 95% CI 0.09–0.75) (P = 0.013). Participants with secondary and above education were more likely to have high knowledge compared to participants with a primary education level (adjusted OR = 2.93; 95% CI 1.28–6.74) (P = 0.011). Participants living in high malaria transmission shehias were more likely to have medium knowledge compared to those living in low transmission shehias (adjusted OR = 2.21; 95% CI 1.29–3.79) (P = 0.004).

### High compared to low knowledge

Participants with secondary and above education were more likely to have high malaria knowledge compared to participants with only primary education 3.45 (adjusted OR = 3.45; 95% CI (1.34–8.89) (P = 0.010). Participants with middle-income were more likely to have high malaria knowledge compared with participants with low-income (adjusted OR = 2.7; 95% CI 1.44–5.09) (P = 0.002).

## Discussion

This study aimed at understanding the levels of malaria knowledge in Zanzibar to provide recommendations to reinforce and scale-up targeted SBC to improve knowledge on the cause, symptoms, and prevention of malaria in the community. Generally, this study showed an increase of malaria knowledge of which 93% of participants correctly identified a mosquito bite as the cause of malaria compared to the 85.4% reported in 2017 KAP study that was conducted in Zanzibar, 81% of the respondents correctly associated fever with malaria compared to the 65.2% reported in 2017 KAP study [21], and the majority reported that an insecticide-treated bed net was one of the best ways to prevent malaria.

As has been documented in other studies, age, transmission level, education, wealth index and employment status were significantly associated with knowledge on malaria [25, 26].

Participants aged ≥ 50 years were more likely to have low malaria knowledge. Although there are few examples that have reported on the relationship between age and malaria knowledge, a study conducted in Malawi suggested that lower age (15–19 years) was associated with low malaria knowledge [14]. Possible explanations for low malaria knowledge among participants ≥ 50 years compared with < 30 years in this study might be less exposure to various sources of health information through various channels including social media primarily targeting the younger generation. Participants with secondary and higher education levels were more likely to have higher knowledge on malaria compared to those with primary education and without formal education. Previous studies have also demonstrated that higher educational attainment was one of the factors associated with high malaria knowledge. A similar study conducted in Rufiji, Tanzania reported that higher education level and the age group 30–49 were significantly associated with higher malaria knowledge [27]. Similar findings were documented in Nigeria [17, 28], Burkina Faso [18], Tanzania [29], and Bangladesh [30]. Another study that investigated correlates of maternal education and childhood malaria infections concluded that children belonging to women with some primary education had a 4% lower chance of being malaria positive, while maternal education beyond primary school was significantly associated with an 8% reduction in malaria prevalence among children under 5 years old [31].

This analysis indicates that individuals with middle-income wealth had higher malaria knowledge compared to those with low-income wealth. Similar findings have also been documented in previous studies. In Madagascar, one study showed that both the mother’s education and household wealth strongly influenced knowledge about and efforts to prevent and treat malaria. This analysis also revealed that the prevalence of malaria among children aged 6–59 months was determined by household wealth [32]. Another study conducted in Bangladesh suggested that economics plays a role in malaria. In this example, families with less wealth had a higher prevalence of malaria, likely influenced by construction materials used in their homes [30].

This study showed that participants who were formal employees had higher malaria knowledge compared with other occupational status (i.e., fishing and farming). This observation was similar to previous reports, one of which was conducted in Uganda and found the factors associated with knowledge on malaria prevention methods were age, employment status, education, income and having heard malaria message in the previous 12 months [33].

Although statistically insignificant, contrary to other studies, these findings suggest that females have higher knowledge on malaria compared to males [34, 35]. While malaria affects both men and women, gender roles and gender dynamics in Zanzibar can give rise to different vulnerabilities to malaria. Gender often intersects with other factors, such as income and education, which contribute to poor malaria outcomes [36]. Despite the findings in this study, females often lack the freedom to make decisions in their households, including for the prevention and treatment of malaria. The study findings might also suggest an increased need to improve malaria knowledge among men in Zanzibar. Other studies have documented that most of the decisions for health seeking are determined by male-led households. In Kenya, women must often ask their husband for permission to access malaria treatment for themselves and their children [37], and other similar findings were documented in Yemen [38].

## Study limitations

In this study, more women were interviewed as representatives of the heads of household; this was likely due to the absence of men in the households at the time of interviews for various social and economic activities. In addition, interviewing only the head of the household may have biased the findings, as their responses might not accurately reflect the knowledge of other household members. There was no study component that observed participants, and the survey depended on self-report that could have recall bias. Misclassification of participants living in high and low malaria transmission shehia might have resulted from population movement or infection of participants in a location other than the shehia in which they lived. Knowledge is an important factor in increasing malaria care seeking and prevention behaviours however there are other psychosocial and contextual factors that can influence behaviour beyond knowledge [22]. Additional research on a broader range of social and behavioural factors and behavioural outcomes could complement these study findings and contribute to evidence-based social and behaviour change interventions. And finally, an incidence cut off of < 1/1000 and ≥ 1.9/1000 population might have been too small to identify a significant variation on the level of malaria knowledge between high and low incidence shehias. Despite these limitations, the findings in this study can be generalized to Unguja Zanzibar to develop targeted SBC interventions based on the identified knowledge gap.

## Conclusion

Overall, the majority of participants in this survey had a high level of knowledge on the cause, symptoms, and prevention of malaria. Despite observing differences in malaria knowledge between high and low malaria transmission shehias, those participants with low knowledge were older and the population with lower levels of education and income. Tailored SBC interventions to increase malaria knowledge in specific groups observed to have low malaria knowledge might enhance the uptake of malaria prevention and treatment services in the community. Resources can be better allocated by ZAMEP and the partners to target those specific groups to increase their level of malaria knowledge.

## Data Availability

Data from this study can be accessed upon request through the corresponding author who will submit a formal request to the Ministry of Health through the Zanzibar Malaria Elimination Programme.

## Abbreviations

GTS: Global technical strategy
IRS: Indoor residual spraying
KAPB: Malaria Knowledge, Attitude, Practice and Behaviour survey
LLIN: Long-lasting insecticidal nets
SADC: Southern African Development Community
WHO: World Health Organization
ZAMEP: Zanzibar Malaria Elimination Programme

## References

[1] WHO. World malaria report 2021. Geneva: World Health Organization; 2021. https://apps.who.int/iris/handle/10665/350147. Accessed 10 Mar 2022.

[2] WHO. Global technical strategy for malaria 2016–2030. 2021 update. Geneva: World Health Organization; 2021. https://apps.who.int/iris/handle/10665/342995. Accessed 10 Mar 2022.

[3] WHO. World malaria report 2017. Geneva: World Health Organization; 2017. https://apps.who.int/iris/handle/10665/259492. Accessed 10 Mar 2022.

[4] WHO. World malaria report 2020: 20 years of global progress and challenges. Geneva: World Health Organization; 2020. https://apps.who.int/iris/handle/10665/337660. Accessed 10 Mar 2022.

[5] Zanzibar Malaria Elimination Strategic Plan 1V. Zanzibar: Ministry of Health; 2018.

[6] Mission report of the Zanzibar Malaria Elimination Audit. Zanzibar: WHO-AFRO; 2015.

[7] Zanzibar malaria elimination social and behavior change communication (SBCC) strategy 2018–2023. Zanzibar: Ministry of Health; 2018.

[8] WHO. Malaria surveillance, monitoring and evaluation: a reference manual. Geneva: World Health Organization; 2018. https://apps.who.int/iris/handle/10665/272284. Accessed 10 Mar 2022.

[9] Zanzibar Malaria Annual Report 2019. Zanzibar: ZAMEP; 2019.

[10] Zanzibar Malaria Elimination Programme. Annual report. Zanzibar: ZAMEP; 2021.

[11] Zanzibar Mid term review of the malaria strategic plan 1V. Zanzibar: Ministry of Health; 2020.

[12] Baltzell K, Harvard K, Hanley M, Gosling R, Chen I (2019). What is community engagement and how can it drive malaria elimination? Case studies and stakeholder interviews. Malar J.

[13] WHO (2003). Prevention and control of malaria epidemics.

[14] Sixpence A, Nkoka O, Chirwa GC, Milanzi EB, Mangani C, Mathanga DP (2020). Levels of knowledge regarding malaria causes, symptoms, and prevention measures among Malawian women of reproductive age. Malar J.

[15] Hlongwana KW, Mabaso ML, Kunene S, Govender D, Maharaj R (2009). Community knowledge, attitudes and practices (KAP) on malaria in Swaziland: a country earmarked for malaria elimination. Malar J.

[16] Szymona-Pałkowska K, Janowski K, Pedrycz A, Mucha D, Ambroży T, Siermontowski P (2016). Knowledge of the disease, perceived social support, and cognitive appraisals in women with urinary incontinence. Biomed Res Int.

[17] Oladimeji KE, Tsoka-Gwegweni JM, Ojewole E, Yunga ST (2019). Knowledge of malaria prevention among pregnant women and non-pregnant mothers of children aged under 5 years in Ibadan, South West Nigeria. Malar J.

[18] Yaya S, Bishwajit G, Ekholuenetale M, Shah V, Kadio B, Udenigwe O (2017). Knowledge of prevention, cause, symptom and practices of malaria among women in Burkina Faso. PLoS ONE.

[19] Hwang J, Graves PM, Jima D, Reithinger R, Ethiopia MIS Working Group (2010). Kachur SP (2007) Knowledge of malaria and its association with malaria-related behaviors—results from the malaria indicator survey, Ethiopia, 2007. PLoS ONE..

[20] Abrar HT (2017). Community knowledge, attitude and practice about malaria and mosquito biting behavior in Southern Ethiopia. AJBIO.

[21] Zanzibar Knowledge, Attitudes, Practices and Behaviour survey (KAPB) 2017. Zanzibar: Ministry of Health; 2018.

[22] Monroe A, Olapeju B, Moore S, Hunter G, Payne Merritt A, Okumu F (2021). Improving malaria control by understanding human behaviour. Bull World Health Org.

[23] Team RC (2014). R: A language and enviroment for statistical computing.

[24] Jolliffe IT, Cadima J (2016). Principal component analysis: a review and recent developments. Phil Trans A Math Phys Eng Sci.

[25] Ayanore MA, Tetteh J, Ameko A, Axame WK, Alhassan RK, Adoliba Ayanore A (2019). Reproductive-age women’s knowledge and care seeking for malaria prevention and control in Ghana: analysis of the 2016 malaria indicator survey. J Trop Med.

[26] Shayo EH, Rumisha SF, Mlozi MRS, Bwana VM, Mayala BK, Malima RC (2015). Social determinants of malaria and health care seeking patterns among rice farming and pastoral communities in Kilosa district in central Tanzania. Acta Trop.

[27] Spjeldnæs AO, Kitua AY, Blomberg B (2014). Education and knowledge helps combating malaria, but not degedege: a cross-sectional study in Rufiji, Tanzania. Malar J.

[28] Dike N, Onwujekwe O, Ojukwu J, Ikeme A, Uzochukwu B, Shu E (2006). Influence of education and knowledge on perceptions and practices to control malaria in Southeast Nigeria. Soc Sci Med.

[29] Mazigo HD, Obasy E, Mauka W, Manyiri P, Zinga M, Kweka EJ (2010). Knowledge, attitudes, and practices about malaria and its control in rural Northwest Tanzania. Malar Res Treat.

[30] Bashar K, Al-Amin HM, Reza MS, Islam M, Asaduzzaman, Ahmed TU (2012). Socio-demographic factors influencing knowledge, attitude and practice (KAP) regarding malaria in Bangladesh. BMC Public Health..

[31] Njau JD, Stephenson R, Menon MP, Kachur SP, McFarland DA (2014). Investigating the important correlates of maternal education and childhood malaria infections. Am J Trop Med Hyg.

[32] Clouston SAP, Yukich J, Anglewicz P (2015). Social inequalities in malaria knowledge, prevention and prevalence among children under 5 years old and women aged 15–49 in Madagascar. Malar J.

[33] Musoke D, Karani G, Ssempebwa J, Etajak S, Guwatudde D, Musoke M (2015). Knowledge and practices on malaria prevention in two rural communities in Wakiso district, Uganda. Afr Health Sci.

[34] Sharma AK, Bhasin S, Chaturvedi S (2007). Predictors of knowledge about malaria in India. J Vector Borne Dis.

[35] Gender and Malaria. Making the investment case for programming that addresses the specific vulnerabilities and needs of both males and females who are affected by or at risk of malaria. United Nations Development Programme; 2015.

[36] MEASURE evaluation (2017). The importance of gender in malaria data.

[37] Molyneux CS, Murira G, Masha J, Snow RW (2002). Intra-household relations and treatment decision-making for childhood illness: a Kenyan case study. J Biosoc Sci.

[38] Al-Taiar A, Chandler C, Al Eryani S, Whitty CJM (2009). Knowledge and practices for preventing severe malaria in Yemen: the importance of gender in planning policy. Health Policy Plan..

